# Development and Validation of a Rapid, Single-Step Reverse Transcriptase Loop-Mediated Isothermal Amplification (RT-LAMP) System Potentially to Be Used for Reliable and High-Throughput Screening of COVID-19

**DOI:** 10.3389/fcimb.2020.00331

**Published:** 2020-06-16

**Authors:** Minghua Jiang, Weihua Pan, Amir Arasthfer, Wenjie Fang, Liyan Ling, Hua Fang, Farnaz Daneshnia, Jian Yu, Wanqing Liao, Hao Pei, Xiaojing Li, Cornelia Lass-Flörl

**Affiliations:** ^1^Department of Laboratory Medicine, The Second Affiliated Hospital and Yuying Children's Hospital of Wenzhou Medical University, Wenzhou, China; ^2^Shanghai Key Laboratory of Molecular Medical Mycology, Shanghai Institute of Mycology, Shanghai Changzheng Hospital, Second Military Medical University, Shanghai, China; ^3^Center for Discovery and Innovation, Hackensack Meridian Health, Nutley, NJ, United States; ^4^Department of Laboratory Medicine, Pinghu Second People's Hospital, Jiaxing, China; ^5^Department of Laboratory Medicine, Pudong New Area People's Hospital, Shanghai, China; ^6^Department of Laboratory Medicine, Wuxi No. 5 People's Hospital, Wuxi, China; ^7^Department of Dermatology, Affiliated Hospital of Hebei University of Engineering, Handan, China; ^8^Institute of Hygiene and Medical Microbiology, Medical University of Innsbruck, Innsbruck, Austria

**Keywords:** COVID-19, SARS-CoV-2, RT-LAMP, qRT-PCR, diagnostic test

## Abstract

**Objectives:** Development and validation of a single-step and accurate reverse transcriptase loop-mediated isothermal amplification technique (RT-LAMP) for rapid identification of SARS-CoV-2 relative to commercial quantitative reverse transcriptase real-time PCR (qRT-PCR) assays to allow prompt initiation of proper medical care and containment of virus spread.

**Methods:** Primers showing optimal *in-silico* features were subjected to analytical sensitivity and specificity to assess the limit of detection (LOD) and cross-reaction with closely- and distantly-related viral species, and clinically prominent bacterial and fungal species. In order to evaluate the clinical utility, our RT-LAMP was subjected to a large number of clinical samples, including 213 negative and 47 positive patients, relative to two commercial quantitative RT-PCR assays.

**Results:** The analytical specificity and sensitivity of our assay was 100% and 500 copies/ml when serial dilution was performed in both water and sputum. Subjecting our RT-LAMP assay to clinical samples showed a high degree of specificity (99.5%), sensitivity (91.4%), positive predictive value (97.7%), and negative predictive value (98.1%) when used relative to qRT-PCR. Our RT-LAMP assay was two times faster than qRT-PCR and is storable at room temperature. A suspected case that later became positive tested positive using both our RT-LAMP and the two qRT-PCR assays, which shows the capability of our assay for screening purposes.

**Conclusions:** We present a rapid RT-LAMP assay that could extend the capacity of laboratories to process two times more clinical samples relative to qRT-PCR and potentially could be used for high-throughput screening purposes when demand is increasing at critical situations.

## Introduction

A new virus causing pneumonia-like infection, COVID-19, which was found in Wuhan, Hubei Province, China, has caused a serious crisis worldwide (Ma et al., 2020). Almost 2 months after the first report, COVID-19 severe outbreaks were reported in numerous countries and became a public health priority in the world (World Health Organization, Situation Report 48). As of May 24, 2020, COVID-19 cases have been found in 213 countries/regions and infected 5,204,508 patients, 337,687 of whom died (World Health Organization, Situation Report 125). The latest phylogenetic analysis studies designated the etiologic agent of COVID-19, SARS-CoV-2 (Wu et al., 2020).

The virulent nature of this virus and its high rate of transmissibility warrants robust, rapid, sensitive, specific, and quantitative diagnostic tools to supplement clinical symptoms aiding clinicians to confidently rule in and rule out patients. Subsequently, a Chinese group used RNA-based metagenomics next generation sequencing (mNGS) to identify the viral RNA from the clinical samples of two patients (Chen et al., 2020). However, the requirement for advanced technology and skilled personnel and long turn-around time (24 h) are not feasible for local and referral laboratories. Therefore, a colorimetric loop mediated isothermal amplification, also known as LAMP, was developed to obviate the need for expensive technologies, e.g., real-time PCR and NGS, as well as to shorten the turn-around time to up to 40 min (Zhang et al., 2020). However, swab samples from limited number of patients (*n* = 7) were included for testing (Zhang et al., 2020). Most recently a newer generation of single step RT-LAMP tests were developed to detect SARS-CoV-2, but these assays were not validated with real clinical samples obtained from COVID-19 positive patients (Lamb et al., 2020; Park et al., 2020). Therefore, we developed a sensitive, specific, and rapid RT-LAMP assay and its performance was challenged by an extensive number of confirmed COVID-19 (*n* = 47) and negative patients (*n* = 213) relative to qRT-PCR assays approved by the National Medical Products Administration (qRT-PCR NMPA). Although our assay was not developed to be quantitative, our assay was proved to be a rapid and reliable diagnostic tool that potentially could be deployed for high-throughput screening applications in referral and local laboratories.

## Materials and Methods

### Target Selection

According to World Health Organization and *Guidelines for prevention and control of Covid-19 (Fourth Edition)* issued by National Health Commission, open reading frame 1ab (ORF1ab) or nucleocapsid protein (N) were recommended for designing diagnostic assays detecting SARS-HCoV-2 from clinical samples (World Health Organization, 2020). Therefore, ORF1ab and N sequences of SARS-CoV-2, its close coronavirus species (HCoV-NL63, HCoV-OC43, HCoV-229E, and HCoV-HKU1), and other viral or bacterial species, namely Adenovirus, Respiratory syncytial virus A, Human parainfluenza virus 2, Human parainfluenza 3 virus, H1N1 influenza virus, H5N1 influenza virus, H7N9 influenza virus, H9N2 influenza virus, Mycoplasma pneumonia, and Influenza B virus, were downloaded from GenBank (https://www.ncbi.nlm.nih.gov/genbank/) to select the most specific target region. Genenious v11.1.14 was used for alignment analysis and to find the most specific region for designing LAMP primers. LAMP Designer (PREMIER Biosoft International, San Francisco, CA) was used for primer design. Designed primers were subjected to BLAST (https://blast.ncbi.nlm.nih.gov/Blast.cgi) and the specific candidates (N gene) were used for analytical sensitivity and specificity testing (Table 1 and Appendix Figure 1). Primers were synthesized by Sangon Biotech Co., Ltd. (Shanghai, China).

**Table 1:** Primers and probes successfully detected SARS CoV-2.

**Target loci**	**Primer name**	**Primer sequence**
Nucleocapsid protein	nCoV-N-F3	CCAGAATGGAGAACGCAGTG
	nCoV-N-B3	CCGTCACCACCACGAATT
	nCoV-N-FIP	AGCGGTGAACCAAGACGCAGGGCGCGATCAAAACAACG
	nCoV-N-BIP	AATTCCCTCGAGGACAAGGCGAGCTCTTCGGTAGTAGCCAA
	nCoV-N-LF	TTATTGGGTAAACCTTGGGGC
	nCoV-N-LB	TTCCAATTAACACCAATAGCAGTCC

### Analytical Sensitivity and Specificity Testing

Since SARS-CoV-2 is not allowed to be cultured in our P2 lab, we performed analytical sensitivity and specificity testing by using pseudotyped SARS-CoV-2 assay system containing ORF1ab part sequence, N gene and E gene (DAAN gene Co. Ltd, Guangzhou, China), to mimic the real virus. RNA of pseudotyped virus was extracted using EZ-10 Spin Column Viral Total RNA Extraction Kit (Sangon Biotech Co., Ltd. Shanghai, China). Serial dilutions with the magnitude of log10 containing 50 × 10^6^ pseudovirus/ml to 50 × 10^0^ pseudovirus/ml were performed to determine the limit of detection (LOD). Serial dilution testing was performed in both RNase/DNase free molecular grade water and sputum sample collected from a COVID-19 negative healthy individual. Reproducibility of our LAMP assay (linearity = *R*^2^-value) was assessed by separate serial dilution testing on three occasions, each performed in duplicate. Signal intensity and the time to obtain amplification curves were recorded and *R*^2^ ≥ 0.98 were considered reliable amplification.

Specificity testing included nucleic acid of various virus or bacteria (Bdsbiotech Co. Ltd, Guangzhou, China). Moreover, Genomic DNA from HeLa cells (TechStar Co. Ltd, Jiangsu, China) and clinically prominent bacteria or fungal species, including *Staphylococcus aureus, Mycobacterium tuberculosis, Legionella pneumophila, Candida albicans, Candida glabrata, Candida tropicalis, Aspergillus fumigatus, Cryptococcus neoformans* were used for specificity testing (provided by Shanghai Institute of Medical Mycology, Shanghai Changzheng hospital). LAMP incubation time was set to 60 min so as to detect both limit of detection and cross-reactivity (LAMP conditions are mentioned in clinical evaluation section). The reaction endpoint time was set in a way to detect the lowest possible copy number of virus without any cross-reaction.

### Evaluating LAMP Assay Tolerance Against Wide Range of Inhibitors

Clinical samples obtained from patients contain a wide range of inhibitors impairing the efficacy of diagnostic assay. Therefore, the tolerance of our LAMP assay was assessed when 500 copy/ml of simulated viral particles were mixed with human blood, mucin, β-adrenergic bronchodilator, Tamiflu, dexamethasone, adrenaline (Appendix Figure 2).

### Clinical Validation

Clinical validation engaged two clinical centers, namely The Second Affiliated Hospital and Yuying Children's Hospital of Wenzhou Medical University, Wenzhou, China (Center 1), and the Wuxi Infectious Diseases Hospital, Wuxi, China (Center 2). Each center used a different qRT-NMPA assay as a gold standard technique. SARS-CoV-2 kit from Shanghai BioGerm Medical Biotechnology Co. Ltd. (NMPA approval number 20203400065, with LOD of 1,000 copies/ml, Ct cut-off 38), and a kit from DAAN Gene Co., Ltd (NMPA approval number 20203400063, with LOD of 500 copies/ml, cut off Ct value of 40) were used in center 1 and center 2, respectively. Positive patients were divided into two groups by physicians, namely suspected and confirmed. Those suspected were isolated and all became positive. The ethics committees of both centers approved the study. Emergency patients (outpatients) with fever of unknown origin or inpatients diagnosed as COVID-19 or other diseases were enrolled and samples such as sputum, nasopharyngeal swabs and tears were used for evaluation. ABI 7500 RT-PCR systems were used for amplification and data analysis in both centers.

The final LAMP reaction was 25 μl and contained 21.9 μl buffer solution (20 mM Tris-HCl pH 8.8, 10 mM (NH_4_)2SO_4_, 120 mM KCl, 2 mM MgSO_4_, 0.1% Tween 20), 8 U Bst DNA polymerase [New England Biolabs (Beijing) ltd, Beijing, China], 0.5 U AMV Reverse Transcriptase (Takara Bio Inc, Dalian, China), 2 μl RNA template, 1.6 μMFIP/BIP primers, 0.2 μM F3/B3 primers, 0.4 μM LF/LB primers, 7 mM MgSO_4_ (Sangon Biotech Co., Ltd., Shanghai, China), 0.8 M betaine (Sangon Biotech Co., Ltd., Shanghai, China), 1.4 mM each dNTP (Takara Bio Inc, Dalian, China), 0.5 μM SYTO-9 (Invitrogen Trading, Co., Ltd., Shanghai, China). LAMP reactions were incubated at 63°C for 30 min in the ABI 7500 PCR machine and florescence data were collected each minute. RT-PCR and RT-LAMP were performed separately by two technicians, and final results were compared.

## Results and Discussion

**Figure 1:**
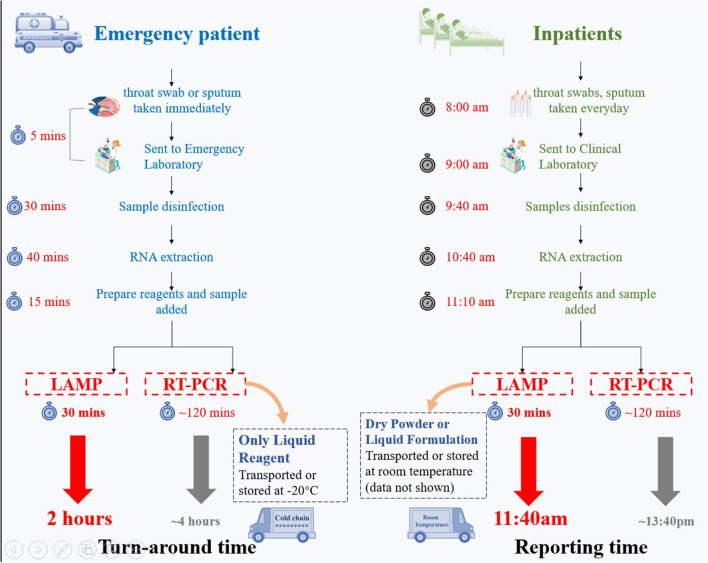
Workflow comparison of our RT-LAMP assay relative to qRT-PCR for emergency cases (outpatients) and inpatients. Our RT-LAMP assay is 2–2.5 times faster than the qRT-PCR assays and can be shipped at room temperature.

**Figure 2:**
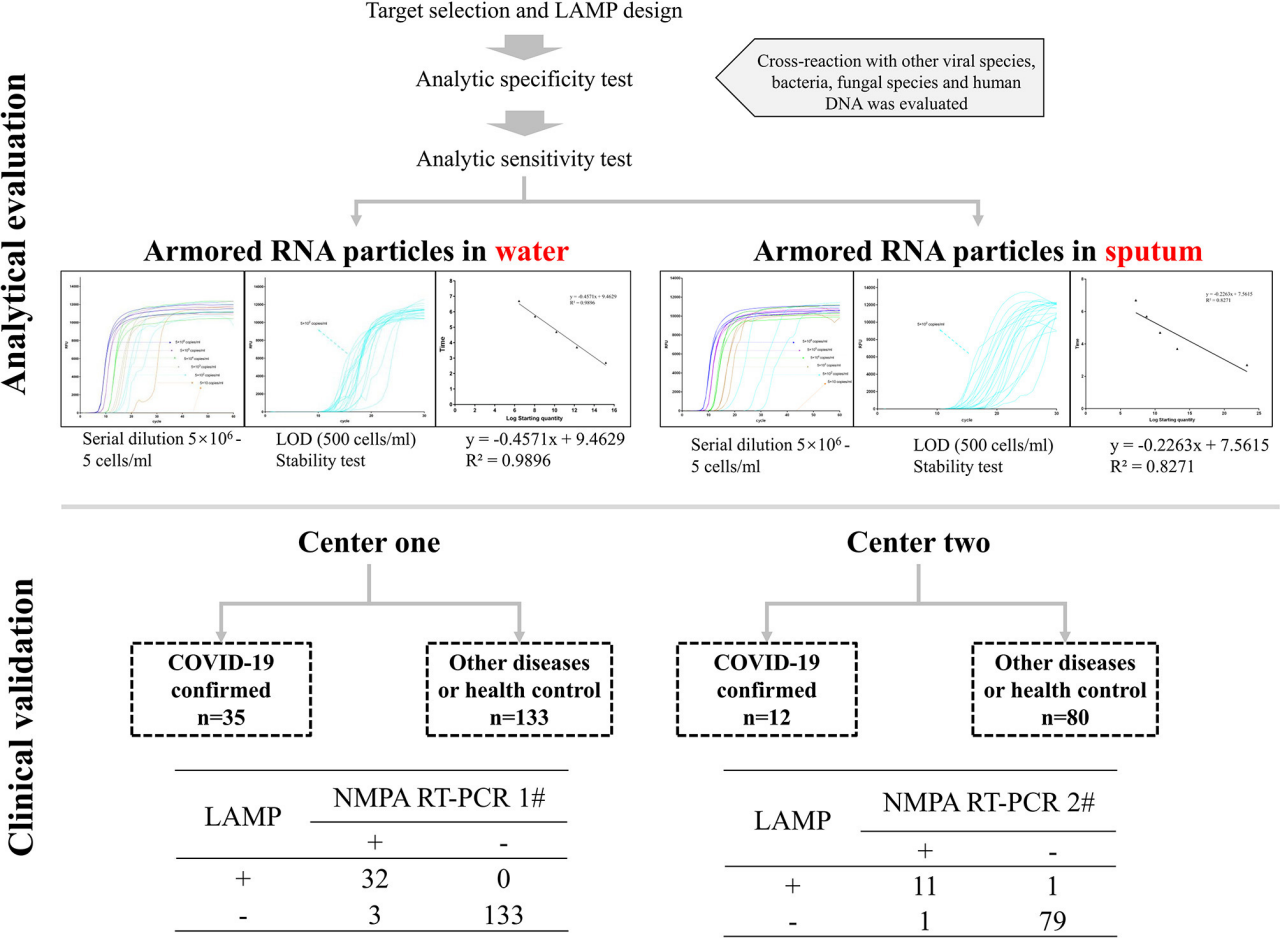
Our assay was comprehensively evaluated at three steps, including *in-silico* analysis, *in-vitro* analytical analysis, and clinical validation.

The whole workflow of our study from *in-silico* analysis to analytical evaluation and clinical validation is depicted in Figure 1. Nine and six LAMP primer systems were designed and evaluated *in-silico*, but only the six primers showed the highest sensitivity and specificity, which used in the next steps (Table 1). Primarily, our assay was meant to be quantitative and it showed an optimal reproducibility when tested in analytical evaluation step using pseudotyped virus diluted in water (*R*^2^ value ~0.99) and sputum sample (*R*^2^ value ~0.83). Analytical sensitivity yielded reliable LOD of 500 copies/ml <30 min regardless of matrix used for serial dilution (Figure 2). Of note, our assay could detect 50 copies/ml, but some replicates showed unstable amplification. Therefore, we considered the LOD of 500 copies/ml. Analytical specificity was 100% when a wide range of closely- and distantly-related viral species, prominent fungal, and bacterial species, and human DNA was used. Moreover, analytical evaluation included a wide range of inhibitors and 500 copies of the simulated viral particles were successfully detected <30 min (Figure 2). In order to evaluate the performance of our assay in clinical setting, we provided our assay and respective instructions to two clinical centers (Figures 1, 2). In total, 168 patients from center 1, including 35 confirmed COVID-19 cases, and 92 patients from center 2, including 12 patients were confirmed COVID-19 cases, were recruited. One asymptomatic patient tested positive by qRT-PCR (Ct values 37) and by our RT-LAMP was categorized suspected by in-charge physician and few days later became positive. Four patients tested positive by qRT-PCR were negative by our RT-LAMP and one patient tested negative by qRT-PCR was positive by our assay (Figure 2 and Appendix Table 1). Subsequently, our RT-LAMP assay showed the sensitivity, specificity, negative predictive value, and positive predictive value of 91.4, 99.5, 98.1, and 97.7%, respectively (Appendix Tables 2, 3). The fact that our assay could not detect four positive patients may be caused by using 2.5 less RNA input (2 μl) relative to qRT-PCR (5 μl). In the future, we will try to use various RNA input volume (5, 8, and 10 μl) to observe if we could obtain a higher sensitivity. Patient 32 (ORF1ab Ct value 17.88, N gene Ct value 18.45) indicates a very high positive may affect signal intensity on LAMP. Further investigations are required by testing highly positive patients. Although our RT-LAMP assay was developed to be quantitative, we could not find any pattern and association between the time to positivity by our RT-LAMP assay and the Ct values reported by qRT-PCR when using clinical samples. Therefore, we considered our assay a qualitative one. This fact will show that the analytical validation should be always accompanied by clinical validation to observe the real capabilities of a given assay and that the results obtained in analytical evaluation step are not always reflected in real-life.

Our assay has several advantages compared to qRT-PCR. First, our RT-LAMP assay is two times faster relative to qRT-PCR (Figure 1) and given the optimal diagnostic features could be used as a reliable screening method in local and referral laboratories to keep up with the increasing demand of suspected patients in critical situations. Secondly, our assay does not need the cold chain and could be shipped at room temperature (Figure 1).

In conclusion, we present a rapid RT-LAMP assay that allows processing 2–2.5 more clinical samples relative to CDC RT-PCR, which is indicative of its capacity to be deployed for high-throughput screening applications in local and referral laboratories.

We admit that our assay does not have the quantitative aspect of qRT-PCR and its sensitivity requires improvement. These two limitations will be the subject of future investigation. Moreover, we will try to use simple and fast nucleic acid extraction procedures (Myhrvold et al., 2018) that only uses heat that will further decrease the turn-around-time.

## Data Availability Statement

The original contributions presented in the study are included in the article/Supplementary Material, further inquiries can be directed to the corresponding authors.

## Ethics Statement

The studies involving human participants were reviewed and approved by Ethics committee of the Second Affiliated Hospital and Yuying Children's Hospital of Wenzhou Medical University. Written informed consent for participation was not required for this study in accordance with the national legislation and the institutional requirements.

## Author Contributions

MJ, WP, and AA participated in primer design, LAMP optimization, data collection, and drafted the manuscript. XL and HP participated in designing this study and revising the manuscript. WF, LL, and HF participated in collecting clinical samples. All authors contributed to the writing of the final manuscript.

## Conflict of Interest

The authors declare that the research was conducted in the absence of any commercial or financial relationships that could be construed as a potential conflict of interest.

## Supplementary Material

Appendix Figure 1 *In silico* specificity of LAMP primers.

Appendix Figure 2 Evaluating LAMP assay tolerance against wide range of inhibitors.

Appendix Table 1 Signal intensity and time corresponded to each serial dilution obtained during three separate experiments. Each experiment was performed in duplicate.

Appendix Table 2 False positive and false negative results reported by our RT-LAMP assay, when compared to qRT-PCR.

Appendix Table 3 Diagnostic feature of our RT-LAMP assay when compared to qRT-PCR.

## References

[B1] Chen L. Liu W. Zhang Q. Xu K. Ye G. Wu W.. (2020). RNA based mNGS approach identifies a novel human coronavirus from two individual pneumonia cases in 2019 Wuhan outbreak. Emerg. Microbes Infect. 9, 313–319. 10.1080/22221751.2020.1725399 32020836 PMC7033720

[B2] Lamb L. E. Bartolone S. N. Ward E. Chancellor M. B. (2020). Rapid detection of novel coronavirus (COVID-19) by reverse transcription- loop-mediated isothermal amplification. medRxiv. 10.1101/2020.02.19.20025155 32530929 PMC7292379

[B3] Ma X. Wang D. Xu W. Wu G. Gao G. F. Tan W. (2020). A novel coronavirus from patients with pneumonia in China, 2019. N. Engl. J. Med. 382, 727–733. 10.1056/NEJMoa2001017 31978945 PMC7092803

[B4] Myhrvold C. Freije C. A. Gootenberg J. S. Abudayyeh O. O. Metsky H. C. Durbin A. F.. (2018). Field-deployable viral diagnostics using CRISPR-Cas13. Science 360, 444–448. 10.1126/science.aas8836 29700266 PMC6197056

[B5] Park G. Ku K. Beak S. Kim S. J. Kim S. I. Kim B.. (2020). Development of reverse transcription loop-mediated isothermal amplification (RT-LAMP) assays targeting SARS-CoV-2. J Mol Diagn. 22, 729–735. 10.1016/j.jmoldx.2020.03.006 32276051 PMC7144851

[B6] World Health Organization (2020). Diagnostic Detection of Wuhan Coronavirus 2019 by Real-Time RT-PCR. Geneva: World Health Organization.

[B7] Wu Y. Ho W. Huang Y. Jin D. Li S. Liu S.. (2020). SARS-CoV-2 is an appropriate name for the new coronavirus. Lancet 6736, 2–3. 10.1016/S1474-4422(19)30448-X 32151324 PMC7133598

[B8] Zhang Y. Odiwuor N. Xiong J. Sun L. Nyaruaba R. O. Wei H. (2020). Rapid molecular detection of SARS-CoV-2 (COVID-19) virus RNA 2. medRxiv. 10.1101/2020.02.26.20028373

