# Supplementary figures and images for: Development and Validation of a Rapid, Single-Step Reverse Transcriptase Loop-Mediated Isothermal Amplification (RT-LAMP) System Potentially to Be Used for Reliable and High-Throughput Screening of COVID-19
Source: Front Cell Infect Microbiol. 2020 Jun 16;10:331. doi: 10.3389/fcimb.2020.00331 (PMC7313420; doi:10.3389/fcimb.2020.00331)

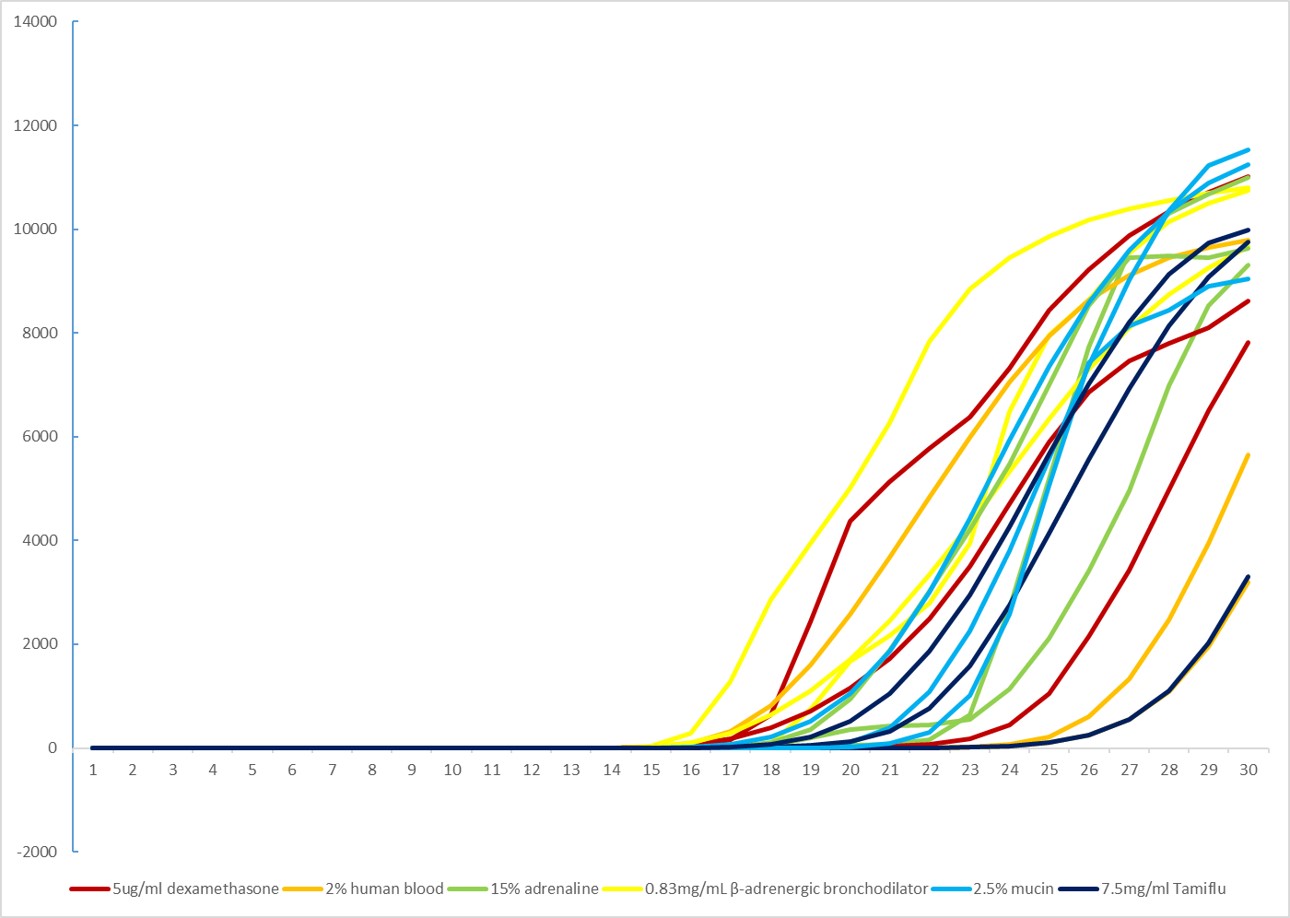

Supplement: Appendix Figure 1 — In silico specificity of LAMP primers. [file Image_1.JPEG]

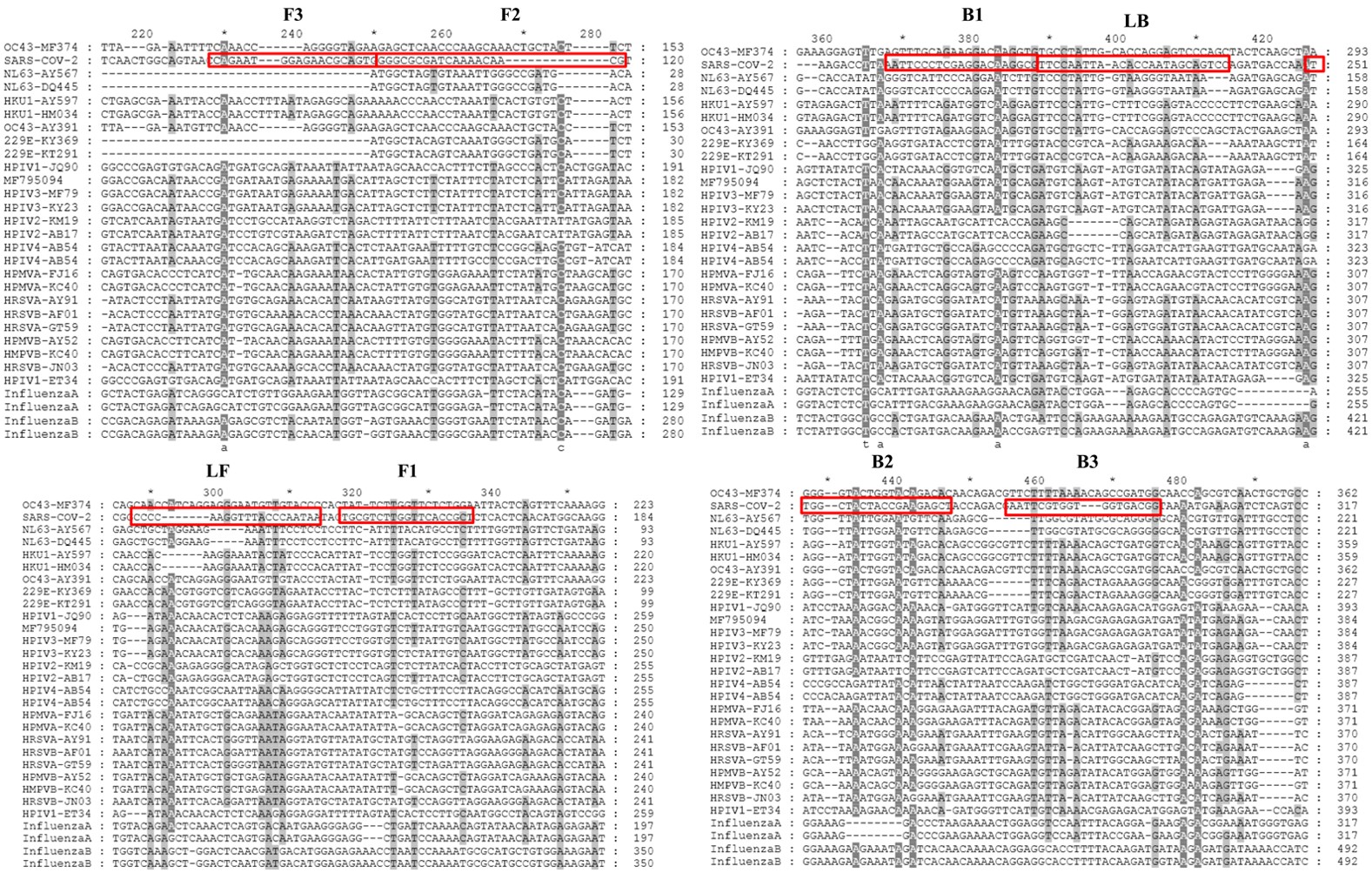

Supplement: Appendix Figure 2 — Evaluating LAMP assay tolerance against wide range of inhibitors. [file Image_2.JPEG]
