# Supplementary material for: Development and Validation of a Rapid, Single-Step Reverse Transcriptase Loop-Mediated Isothermal Amplification (RT-LAMP) System Potentially to Be Used for Reliable and High-Throughput Screening of COVID-19
Source: Front Cell Infect Microbiol. 2020 Jun 16;10:331. doi: 10.3389/fcimb.2020.00331 (PMC7313420; doi:10.3389/fcimb.2020.00331)
Supplement: Appendix Table 1 — Signal intensity and time corresponded to each serial dilution obtained during three separate experiments. Each experiment was performed in duplicate. [file Data_Sheet_1.docx]

**Appendix Table 1.** Signal intensity and time corresponded to each serial dilution obtained during three separate experiments. Each experiment was performed in duplicate.

| **Center 1** | | | | **Center 2** | | | |
| --- | --- | --- | --- | --- | --- | --- | --- |
| **Patient #** | **RT-PCR Ct** | | **RT-LAMP** | **Patient #** | **RT-PCR Ct** | | **RT-LAMP** |
|  | **ORF1ab** | **N gene** | **Signal intensity**  **N gene** |  | **ORF1ab** | **N gene** | **Signal intensity**  **N gene** |
| 1* | 34.26 | 32.97 | 14.89 | 36 | 28 | 30 | 14.2 |
| 2 | 26.31 | 26.18 | 11.13 | 37 | 37 | 37 | 23 |
| 3 | 30.25 | 29.96 | 23.67 | 38 | 19.3 | 22.3 | 7.3 |
| 4 | 34.86 | 35.14 | No Ct value | 39* | 29.5 | 32.1 | 11.7 |
| 5 | 25.86 | 25.14 | 9.97 | 40* | 31.9 | 35.2 | 13.3 |
| 6 | 36.22 | 34.16 | 15.79 | 41* | 26.1 | 28.6 | 12.3 |
| 7 | 22.68 | 24.03 | 11.86 | 42 | 35.2 | 37.9 | no CT value |
| 8 | 34.72 | 29.59 | 16.04 | 43 | 38.5 | 41.2 | 29.2 |
| 9 | 30.41 | 30.72 | 14.42 | 44 | 25.8 | 27.8 | 11.5 |
| 10 | 27.22 | 27.36 | 11.89 | 45 | 28.6 | 31.6 | 13.4 |
| 11 | 28.94 | 29.17 | 12.5 | 46 | No Ct value | No Ct value | 27.5 |
| 12* | 28.77 | 28.53 | 15.23 | 47 | 29.3 | 31.8 | 14.3 |
| 13 | 26.7 | No Ct value | 11.21 | 48 | 23 | 25.3 | 10.6 |
| 14* | 36.24 | No Ct value | 28.42 |  |  |  |  |
| 15 | 33.04 | No Ct value | 13.46 |  |  |  |  |
| 16 | 33.61 | No Ct value | 16.25 |  |  |  |  |
| 17 | 23.31 | No Ct value | 9.76 |  |  |  |  |
| 18* | 25.5 | No Ct value | 10.01 |  |  |  |  |
| 19* | 22.7 | No Ct value | 7.68 |  |  |  |  |
| 20* | 31.28 | 31.33 | 16.89 |  |  |  |  |
| 21* | 27.64 | 27.66 | 13.06 |  |  |  |  |
| 22* | 29.73 | 30.08 | 15.29 |  |  |  |  |
| 23 | 26.31 | 26.18 | 12.58 |  |  |  |  |
| 24 | 30.25 | 29.96 | 20.72 |  |  |  |  |
| 25 | 25.86 | 25.14 | 11.13 |  |  |  |  |
| 26 | 36.22 | 34.16 | No Ct value |  |  |  |  |
| 27 | 22.68 | 24.03 | 11.52 |  |  |  |  |
| 28 | 34.72 | 29.59 | 17.85 |  |  |  |  |
| 29 | 30.41 | 30.72 | 19.28 |  |  |  |  |
| 30 | 27.22 | 27.36 | 14.07 |  |  |  |  |
| 31 | 28.94 | 29.17 | 17.31 |  |  |  |  |
| 32 | 17.88 | 18.45 | No Ct value |  |  |  |  |
| 33* | 34.01 | 33.61 | 14.27 |  |  |  |  |
| 34* | 28.77 | 28.53 | 18.37 |  |  |  |  |
| 35* | 35.01 | 34.61 | 13.21 |  |  |  |  |
| **Mean (95% CI)** | 29.39  (27.80 to 30.97) | 29.05  (27.58 to 30.53) | 14.68  (13.14 to 16.22) |  | 29.35  (25.77 to 32.93) | 31.73  (28.27 to 35.19) | 15.69  (11.27 to 20.11) |

* means swab sample; the others without “*” mean sputum.

**Appendix Table 2**. False positive and false negative results reported by our RT-LAMP assay, when compared to qRT-PCR.

| **Center one** | **LAMP** | **NMPA RT-PCR 1#** | |
| --- | --- | --- | --- |
|  |  | + | - |
|  | **+** | 32 | 0 |
|  | **-** | 3 | 133 |
| **Center two** | **LAMP** | **NMPA RT-PCR 2#** | |
|  |  | + | - |
|  | **+** | 11 | 1 |
|  | **-** | 1 | 79 |
| **Total** | **LAMP** | **NMPA RT-PCR 1-2** | |
|  |  | **+** | **-** |
|  | **+** | 43 | 1 |
|  | **-** | 4 | 212 |

**Appendix Table 3**. Diagnostic feature of our RT-LAMP assay when compared to qRT-PCR.

|  | **Center one** | **Center two** | **Total** |
| --- | --- | --- | --- |
| **Sensitivity** | 91.43% | 91.67% | 91.49% |
| **Specificity** | 100.00% | 98.75% | 99.53% |
| **Accuracy** | 98.21% | 97.83% | 98.08% |
| **Postive predictive value** | 100.00% | 91.67% | 97.73% |
| **Negative predictive value** | 97.79% | 98.75% | 98.15% |
| **Yuden index** | 91.43% | 90.42% | 91.02% |
